# Low-Cost Graphite on Paper Pressure Sensor for a Robot Gripper with a Trivial Fabrication Process

**DOI:** 10.3390/s18103300

**Published:** 2018-10-01

**Authors:** Jarred Fastier-Wooller, Toan Dinh, Van Thanh Dau, Hoang-Phuong Phan, Fuwen Yang, Dzung Viet Dao

**Affiliations:** 1 Griffith School of Engineering and Built Environment, Griffith University, Gold Coast, QLD 4222, Australia; jarred.fastier-wooller@griffithuni.edu.au (J.F.-W.); fuwen.yang@griffith.edu.au (F.Y.); 2 Queensland Micro- and Nanotechnology Centre, Griffith University, Brisbane, QLD 4111, Australia; toan.dinh@griffithuni.edu.au (T.D.); hoangphuong.phan@griffithuni.edu.au (H.-P.P.); 3 Research Group of Environmental Health, Sumitomo Chemical Ltd., Hyogo 665-8555, Japan; dauthanhvan@gmail.com

**Keywords:** graphite on paper, paper switch, resistive pressure sensor

## Abstract

A flexible pressure sensor with a rudimentary, ultra-low cost, and solvent-free fabrication process is presented in this paper. The sensor has a graphite-on-paper stacked paper structure, which deforms and restores its shape when pressure is applied and released, showing an exceptionally fast response and relaxation time of ≈0.4 ms with a sensitivity of −5%/Pa. Repeatability of the sensor over 1000 cycles indicates an excellent long-term stability. The sensor demonstrated fast and reliable human touch interface, and successfully integrated into a robot gripper to detect grasping forces, showing high promise for use in robotics, human interface, and touch devices.

## 1. Introduction

Developing flexible and high sensitivity sensors for robot hands is of interest for accomplishing a wide range of tasks such as object manipulation, articulation, and gesture activities. The requirements for these sensors include but are not limited to (1) high sensitivity and a wide measurement range, (2) fast response and excellent repeatability, (3) soft flexibility and stretchability, and biodegradability, (4) low cost and simplicity for implementation/integration into robot hands. These sensors can range from simple two-state switches to high precision pressure/tactile sensors [1,2,3,4,5]. For example, several studies have successfully demonstrated tactile sensors with the capability of both operating in a tactile and proximity mode for tracking object motion and high-speed hands [6,7].

The working principle of tactile/pressure sensors is typically based on piezoresistive, resistivity, and capacitive effects [8,9,10,11]. The recent development of these sensors is summarised in Table 1. Apart from robot hand applications, these sensors have been applied in power switches, keypads/keyboards, touchscreens, weight scales, pressure control/feedback systems, etc. Scientific investigations have been performed on various alternatives to improve and diversify these methods to provide high performance, yet low-cost alternative sensors. Sensor fabrication can include many expensive/advanced materials, such as carbon nanotube/graphene, and complicated procedures/processes that involve cleanroom facilities and toxic chemicals, as seen in Table 1. The devices described in this table are ordered by the sensing principle to allow for a more relevant comparison.

Environmental issues have been brought to attention, causing a growing interest in the need to develop eco-friendly and biodegradable sensors, for a wide range of applications, including robotics and flexible electronic devices. Paper-based sensory devices have been paid a great deal of attention, owing to their low cost, robustness, biodegradability, and flexibility. More specifically, Graphite-on-Paper (GoP) devices, capable of alleviating many complex fabrication processes, by substituting them with simple pencil drawn structures on a paper substrate, are of high interest for flexible electronic applications. These GoP devices have been successfully investigated in terms of resistors [26], capacitors [27], field effect transistor [28], and chemiresistors for both physical and chemical sensors [29]. Since piezoresistive/resistive pressure sensors have certain advantages in terms of design simplicity, fabrication, and characterisation, GoP resistive pressure sensors could provide further insights into the development of ultra-low cost smart robotics [28,30,31].

In the current work, we present the design, fabrication, and characterisation of a novel ultra-low cost GoP resistive pressure sensor with a very simple fabrication method. The sensor showed an exceptionally fast response time compared to that of other literature of 0.4 ms, with excellent long-term stability, after a thousand cycles of testing. Our sensor has a much faster response time, which is at least one order of magnitude faster than that of other pressure sensors, Table 1. In addition, while pressure sensors manufactured by classical mixing process have been reported with the occurrence of sensitivity degradation under dynamic loading or highly non-linear response [32,33,34], our sensors exhibited excellent long-term stability, repeatability, and no damage when overloading. Successful demonstration of the sensor as tactile feedback for both human and robotic applications shows the potential of the sensor as an ultra-low cost rapid prototyping device, requiring no expensive materials or equipment, and no solvents. The performance of our sensor is comparable to that of numerous sensors employing advanced materials, showing that it has potential uses in robot hands and other versatile fields whose facilities have inadequate funding or lack of access to advanced fabrication processes.

## 2. Sensor Principle and Design

**Figure 1:**
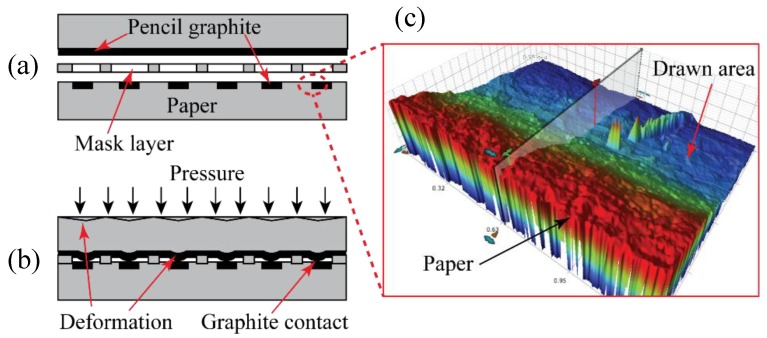
(**a**) Concept sketch of sensing mechanism with no applied pressure, (**b**) concept sketch of the sensing mechanism with applied pressure, and (**c**) 3D surface profile of GoP devices.

Figure 1a,b shows a schematic cross-sectional sketch of the sensor mechanism. Figure 1c shows a 3D surface profiling image of the GoP in which we can see how pinpoint pressure exerted on the paper during the drawing process causes the drawn areas to deform. Both the paper and the graphite areas share a rough surface area, leading to a pressure dependent contact resistance between the graphite-shaded layers. Pressure is applied perpendicular to the sensor, until the paper deforms, allowing the two graphite layers to make contact through the gaps in the mask, seen in Figure 1b. As pressure increases, the contact area between the graphite layers increases. Increased contact between the graphite layers causes a reduction in the overall resistance between terminals causing an increased measured electrical current.

**Figure 2:**
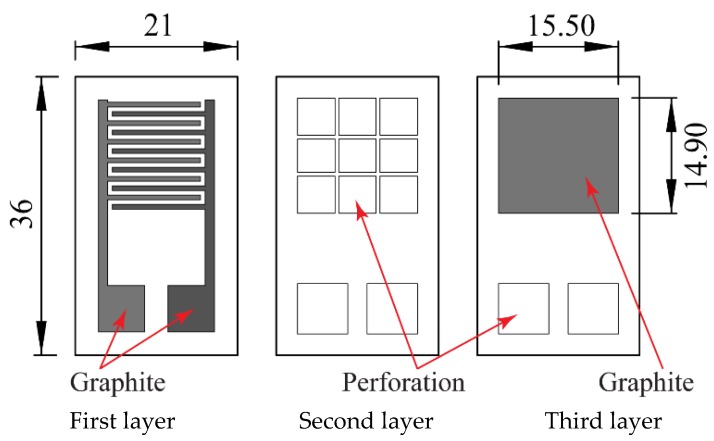
Three main layers of the sensor.

A complete and more detailed image of the proposed sensor design can be seen in Figure 2.

## 3. Fabrication of the Sensor

**Figure 3:**
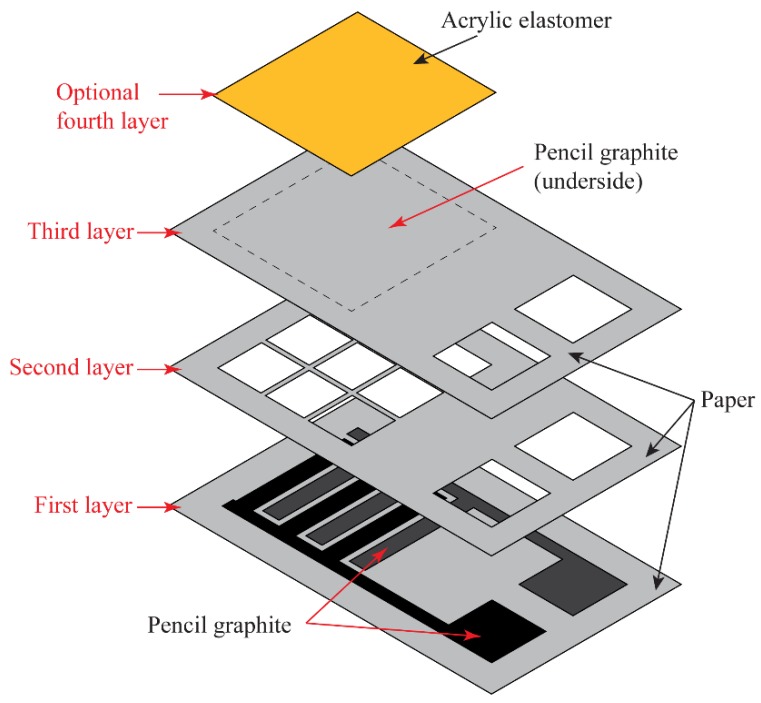
Assembly of the sensor.

The presented sensor is fabricated using elementary and very low-cost methods. The sensor frame is first printed on to a sheet of paper (80 gsm Staples A4) using any office or home printing device. The outlined areas for graphite shading are filled-in using a graphite pencil (5B Faber-Castell). Using a scalpel/Stanley knife, the sensor outlines and windows are delicately cut and prepped for assembly. Each layer is aligned and held together with tape (Keji Clear Adhesive). To provide a more elastic sensing surface, a 500 μm thick acrylic elastomer layer (3M VHB Acrylic Foam Tape) can be adhered to the sensing area of the sensor, as seen in Figure 3, to provide a softer interface (ergonomic feedback) when pressing down on the sensor. The second layer is used as a spacer for the two sensing elements in the device, allowing an ultra-high sensitivity to the initial touch, allowing the device to be used as a switch. The manual fabrication process using a pencil-drawn approach could lead to the variability of the dimensions of the sensing elements. To better control the size and thickness of graphite layers, we recommend that a precisely controlled XY stage can be implemented.

## 4. Results and Discussion

### 4.1. Material Properties

**Figure 4:**
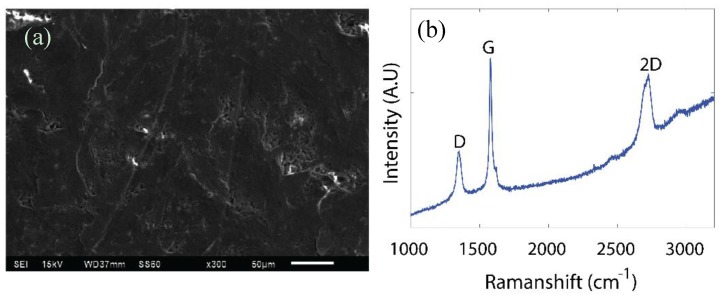
(**a**) Scanning Electron Microscopy (SEM) image of GoP, (**b**) Raman spectrum of GoP.

The deposition of graphite on a porous paper substrate can be seen in Figure 4a. Three main peaks were observed in the Raman spectroscopy, presented in Figure 4b, at the wavenumbers of 1350, 1580, and 2725 cm^−1^, respectively corresponding to the D, G, and 2D bands of the graphite material. The intensity of the G-band was much larger than that of the D-band, which was indicative of the material’s high quality [35].

### 4.2. Switching

**Figure 5:**
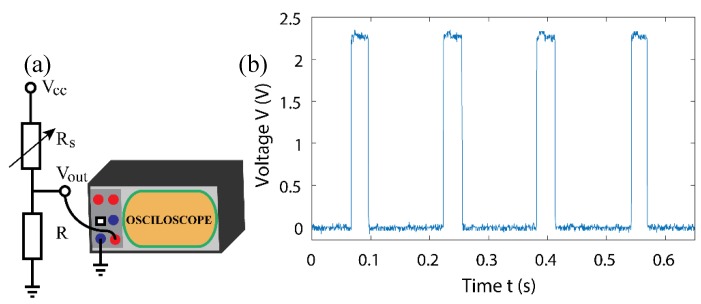
(**a**) Experimental setup (V_cc_ = 5 V and R = 10 MΩ), (**b**) switching cycles of sensor.

A simple voltage divider circuit was implemented with a constant voltage source (Bench Power Supply, E3631A) and a Keysight Oscilloscope (MSO-X 3104A), for measurement. Schematic configuration for this experiment can be seen in Figure 5a. Pressure impulses were applied to the sensor by repeatedly tapping down on it gently, with a single index finger. It can be seen in Figure 5b that the sensor has a steep increase in conductivity, under the applied low pressure. At a low-pressure range, the sensor functions as a switch, where the OFF stage (i.e., no touch) is approximately 0 V and the ON stage is a much higher level of approximately 2.3 V. The difference of the touching force is very small, compared to the level of the signal at the ON stage. In this case, the touching force is expected to generate a pressure of less than 100 kPa. Therefore, the GOP device is suitable to functions as a switch, at a low pressure (e.g., below 100 kPa).

### 4.3. Pressure Calibration

**Figure 6:**
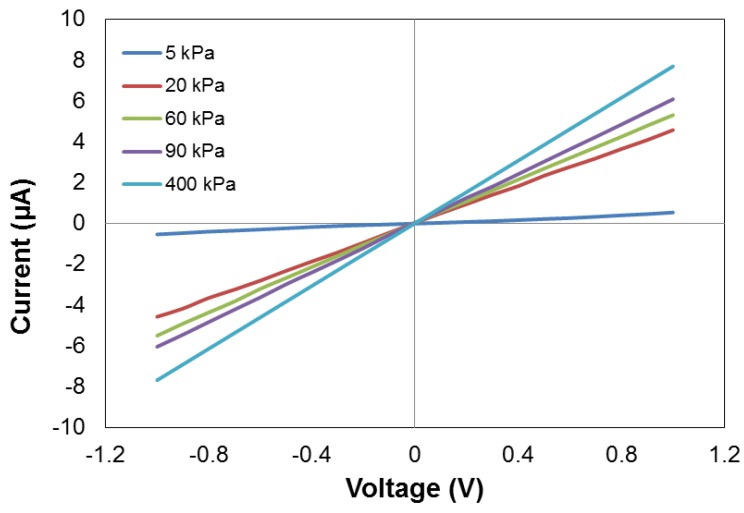
Current-voltage (I–V) characteristics of the sensor under different applied pressures.

The sensors pressure capabilities were calibrated and tested using a Keysight USB Modular Source Measure Unit (U2722A) and an Instron Universal Testing Machine (Model 3367). Measurements were taken from both instruments, simultaneously, to obtain a reliable force/time and resistance/time values. We have measured the current–voltage (I–V) curve of the device, under different pressures, as presented in Figure 6. It was evident that all the I–V curves showed good linear characteristics, for the applied voltage, from −1 V to 1 V, indicating the good Ohmic contacts of the device. Consequently, the performance of the device was independent of the applied voltage. This also suggested that the quantum tunnelling was not dominant in our device [36,37]. 

**Figure 7:**
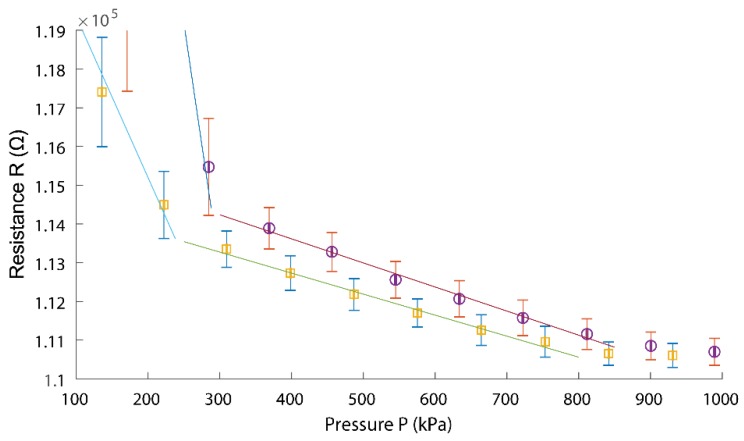
Calibration results where the x–y standard deviation around the mean is represented as error bars, circle indicates compression, and square indicates decompression.

The measured force and resistance values have been aligned and combined below, in Figure 7. Pressure was applied to the sensor using a piece of precision cut 15 × 15 × 3 mm rubber (Goodyear natural rubber insertion), as such all force measurements have been appropriately converted to pressure.

Based on a line of best fit, calibration showed a sensitivity of −5.4 × 10^−5^ kPa^−1^ between 300 and 850 kPa, under compression, and of −4.8 × 10^−5^ kPa^−1^ between 250 and 800 kPa, when released. The overall sensitivity of the sensor was, thus, referred to as approximately −0.5 × 10^−4^ kPa^−1^, in its main sensing region, from 250–800 kPa. Lower pressure regions of the sensor could be characterised as −9.1 × 10^−4^ kPa^−1^, between 100 and 300 kPa, under compression, and of −3.5 × 10^−4^ kPa^−1^, between 100 and 250 kPa when released. The sensitivity of our sensors was comparable to that of other pressure sensors [25,38,39,40]. In addition, the dedicated structure (very thin paper making contact window) provided an ultrafast sensor response time.

Physical connections were formed between the rough surfaces of the graphite layers, during compression. Due to the rough surface, these formed connections could stay connected, at lower pressure, while being decompressed, showing lower overall resistance in decompression. There were two distinct regions of sensitivity, the heightened sensitivity of the 100–300 kPa region could be attributed to the initial contact of the graphite-sensing areas, while the pressure range of 300–800 kPa was no longer affected significantly, by the new contacts.

**Figure 8:**
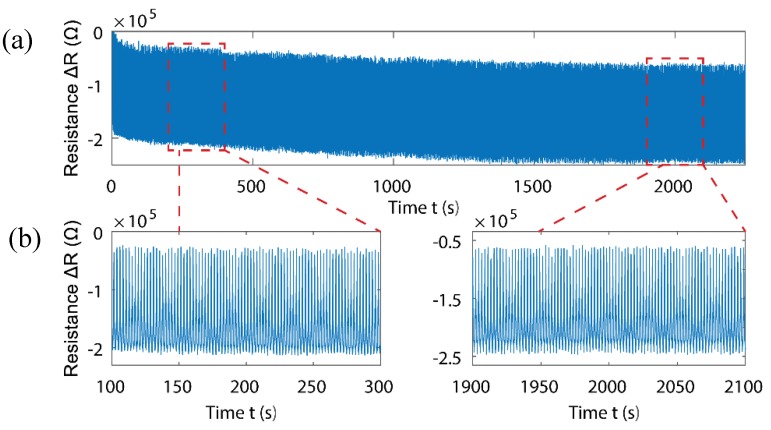
(**a**) Complete 1000-cycle test, (**b**) two graphs show more detailed start and end of cycle test.

Cycle testing was performed on the sensor by applying 10–100 N of force (≈21–210 kPa of pressure) on the sensor in intervals of approximately 2.2 s. Figure 8a,b shows the change in resistance for 1000 cycles over the duration of 37.5 min. The repeatability of the sensor over 1000 cycles, shows an excellent long-term stability. However, due to the ultra-low-cost materials and the simplicity of fabrication of the GoP, each sensor could be used in short-term service, for rapid prototyping.

The calibration results have been performed using an ohmmeter, with no reference to the influence of a source voltage to the system, as resistance measurements were taken without a source voltage. In addition, the linear current–voltage characteristics of the sensor, at different applied pressures, would suggest the independence of the sensor performance on the sourcing voltage. For future studies, the repeatability, drift, and hysteresis errors at different voltages could be further investigated to assess the overall impact of the source voltage on the device and its sensing characteristics.

### 4.4. Response and Relaxation

A simple voltage divider circuit was implemented, with a constant voltage source, to measure the response rate, performed using a Keysight Oscilloscope (MSO-X 3104A). Schematic configuration and method for this experiment was the same as used to test the sensors response to touch, as shown in Figure 5a. Fast impulses were applied to the sensor using a single index finger and response times, as fast as 50 µs, were observed on the oscilloscope, showing how effective each impulse could be.

**Figure 9:**
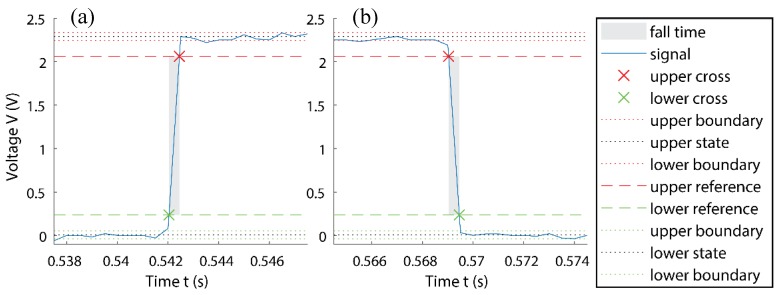
(**a**) Measured response time, (**b**) measured relaxation time.

However, the exported data had clipped time intervals of 0.5 ms, effectively, limiting the calculable response, as a lot of data was emitted by the oscilloscope, when saving to the USB. Based on the recorded data, an excellent rise and fall time of ≈0.4 ms could be seen in Figure 9a,b, calculated and presented in MATLAB, using standard *risetime()* and *falltime()* functions. The reaction behaviour of the sensor included the mechanical response time and the reaction rate of the graphite. The mechanical response was the deformation of a very thin paper layer, making the contact window, and the reaction rate of graphite was fast. Altogether the sensor featured a response as fast as 40 µs, corresponding to a bandwidth of 2.5 kHz. This bandwidth satisfied the practical applications of robot hands, where the frequency bandwidth was typically lower than 2.5 kHz. The presented sensor was, therefore, capable of at least a ≈2.5 kHz switching-frequency, based on the slower response of the recorded data. This indicated the potential of using our sensor for high-speed robot hands and other high frequency applications.

### 4.5. Use in Robotic Touch Feedback Sensing

**Figure 10:**
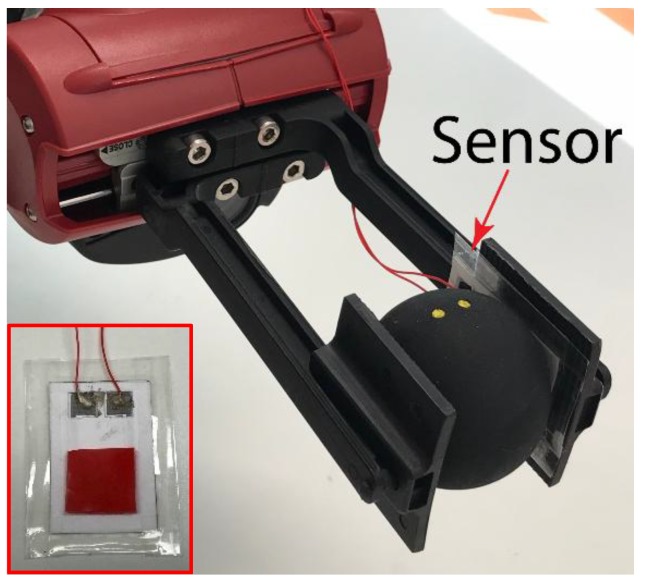
Sensor integrated into a robot gripper, (**inset**) fabricated sensor with optional fourth layer for better grip.

**Figure 11:**
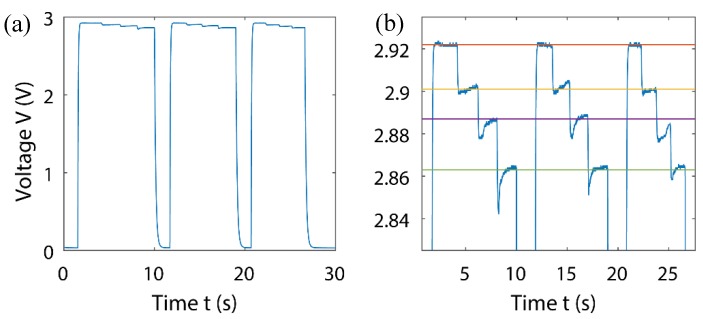
(**a**) Contact of sensor to ball, (**b**) close-up showing four distinct levels of applied force.

The sensor was integrated into a Baxter robots stock parallel grippers’ fingertip and used to pick up and hold a squash ball (Dunlop Pro), as shown in Figure 10. For demonstration, a simple voltage divider circuit, with a passive low-pass filter (50 Hz cut-off) was used in conjunction with a Tiva microcontroller (Texas Instruments, TM4C123GH6PM). Custom computer software was used to poll the microcontroller and record the measurement data. The grippers moving force, holding force, velocity, and position were controlled using the computer software. The ball was deformed when gripped, and then held onto, before being released slowly, in stages, as seen below in Figure 11a.

A quick rise in the output voltage was observed in Figure 11a, when the gripper first contacts the ball. Step decreases of the output voltage, detailed in Figure 11b, corresponded to the decrease in the applied pressure to the ball. The change in pressure was controlled by setting the gripping speed and modifying the desired displacement value, as the built-in controller did not allow for repeatable precision control; hence the four distinct steps were not uniform. The ball had a varying area of contact with the sensor. It was not possible to show the calibrated pressure conversions, without knowing the changing contact area of the ball, due to the performed calibration being dependent only on force, as area was non-variable. Spikes in measurements were noticed when the gripper was in motion, this could be due to the elastic properties of the ball or software implemented safety restrictions. Despite this, a fast response and clear change in signal output indicates the viability of integrating our sensor in robot grippers, providing a basis for further investigation.

It has been shown that the first stage (Figure 11a) was for touch detection, and the second stage (Figure 11b) was for gripping force/pressure measurement. When implemented with digital control, it was possible for the control unit to compromise with the calibration curves. Further simplification of the calibration process could, therefore, be performed using these control units/microcontrollers and a set of known weights.

## 5. Conclusions

We successfully fabricated an ultra-low-cost, biodegradable, and fast response multi-purpose resistive pressure sensor. A rudimentary and effective fabrication process has been presented without the use of any solvent-based materials. The sensor showed a fast response time of 0.4 ms and a sensitivity of −5%/Pa, in the range of 300–800 kPa. The sensor was capable of high-performance, as a switch for human touch sensing as well as a pressure sensor for robotic tactile feedback. Our sensor is a versatile, resistive, pressure sensor, capable of detecting touch motion in near real-time, demonstrating a strong feasibility of using this sensor for tactile sensing, touch displays, and wearable applications. Due to the sensor’s ultra-low-cost and rapid prototyping method with standard household tools, it is possible that the sensor can be used in institutes, such as in education centres, where more advanced facilities or funding are not available.

## Figures and Tables

**Table 1:** Performance of pressure sensors in literature.

Pressure Sensor	Materials	Design and Implementation Cost	Sensitivity	Range	Response/Relaxation	Solvent	Ref.
Capacitive	sparkling graphene block	automatic egg beater, freeze dry, annealing	229.8 kPa^−1^ 26.86 kPa^−1^	0–0.12 kPa 0.4–1.0 kPa	≈1085 mm s^−1^ “recovery speed”	Yes	[2]
Capacitive	SBS AgNP Composite-coated Kevlar Fibre	SBS coating, Ag precursor absorption, precursor reduction	0.210 kPa^−1^ 0.064 kPa^−1^	<2 kPa >2 kPa	≈40 ms ≈10 ms	Yes	[6]
Capacitive	Au-electroplated planar coil. Si/glass substrates	dissolved-wafer process	1580 ppm/mmHg	0–50 mmHg	120 kHz/mmHg	Yes	[12]
Capacitive	MG/PU composite film	solution compounding method	0.274 kPa^−1^	0–0.2 kPa	---	Yes	[13]
Capacitive	PDMS coated graphite on paper	paper, pencil, PDMS	0.62 kPa^−1^	<2 kPa	200 ms rise 400 ms fall	Yes	[14]
Capacitive	PDMS, CPDMS, Ecoflex	photolithography, micro-contact printing, spin-coating, thermal curing	0.42 Pa^−1^	0–1.2 mPa	---	Yes	[15]
Capacitive	Au nanowire coated tissue paper, PDMS	dip coating/drying, PDMS, PDMS patterned with integrated electrodes	1.14 kPa^−1^	5 kPa	<17 ms	Yes	[16]
Piezo-resistive	graphene	CVD sputtering system	−0.24kPa^−1^ 0.039kPa^−1^	0.3–200 Pa 700+ Pa	>40 ms	Yes	[1]
Piezo-resistive	Au@PU	ion sputtering	0.059 kPa^−1^	0–5 kPa	9 ms	No	[17]
Piezo-resistive	sponge@CNTs@Ag NPs	“dip and dry” technique	2.12 kPa^−1^ 9.08 kPa^−1^	2.24–11 kPa 11–61.81 kPa	---	Yes	[18]
Piezo-resistive	CB@PU sponges	water-based LBL assembly	0.068 kPa^−1^ 0.023 kPa^−1^ 0.036 kPa^−1^	≈0–2.3 kPa 2.3− ≈ 10 kPa ≈10− ≈ 16 kPa	<20ms	Yes	[19]
Resistive	VACNT/PDMS composite	CNT (T-CVD) sandblasting, etc.	~0.3 kPa^−1^ ~0.05 kPa^−1^	0–0.7 kPa 0.7–2 kPa	≈162 ms ≈108 ms	Yes	[3]
Resistive	Au-patterned polydimethylsiloxane membrane	MEMs process (PR, deposition, etc.)	0.23 kPa^−1^	0–6.7 kPa	≈200ms	Yes	[20]
Resistive	graphene Porous Network Structure and PDMS	PDMS infiltration Ni etching	0.09 kPa^−1^	<1000 kPa	≈100 ms rise ≈80 ms fall	Yes	[21]
Resistive	graphene foam and PDMS	vacuum-assisted dip-coating reduction etching	≈0.6 kPa^−1^ ≈0.8 kPa^−1^ 60 kPa^−1^	0–200 kPa 200–500 kPa 500+ kPa	>10s	Yes	[22]
Resistive	graphene-wrapped PU sponges	RGO-PUS-HT-P sponge	---	9+ Pa	---	Yes	[23]
Resistive	elastic microstructured conducting polymer	---	≈7.7–41.9 kPa^−1^ <0.4 kPa^−1^	<100 Pa >1 kPa	≈50 ms	Yes	[24]
Resistive	CNT/polymer	Chemical vapour deposition, polymer tape	0.15–0.67 Pa^−1^	0–60 kPa	100 ms	Yes	[25]
Resistive	graphite on paper	Paper, Pencil, office tape	≈−0.35 Pa^−1^ ≈−0.05 Pa^−1^	100–250 kPa 300–800 kPa	≈0.4 ms	No	This
